# Specific Cell Targeting Therapy Bypasses Drug Resistance Mechanisms in African Trypanosomiasis

**DOI:** 10.1371/journal.ppat.1004942

**Published:** 2015-06-25

**Authors:** Juan D. Unciti-Broceta, José L. Arias, José Maceira, Miguel Soriano, Matilde Ortiz-González, José Hernández-Quero, Manuel Muñóz-Torres, Harry P. de Koning, Stefan Magez, José A. Garcia-Salcedo

**Affiliations:** 1 Unidad de Enfermedades Infecciosas y Microbiología, Instituto de Investigación Biosanitaria ibs.GRANADA, Hospitales Universitarios de Granada/Universidad de Granada, Granada, Spain; 2 Instituto de Parasitología y Biomedicina “López-Neyra” (IPBLN-CSIC), PTS Granada, Armilla, Spain; 3 Centro Pfizer-Universidad de Granada-Junta de Andalucía de Genómica e Investigación Oncológica (GENYO), PTS Granada, Granada, Spain; 4 Departamento de Farmacia y Tecnología Farmacéutica, Facultad de Farmacia, Universidad de Granada, Granada, Spain; 5 Departamento de Agronomía, Universidad de Almería, Almería, Spain; 6 Unidad de Metabolismo Óseo, Instituto de Investigación Biosanitaria ibs.GRANADA, Hospitales Universitarios de Granada/Universidad de Granada, Granada, Spain; 7 Institute of Infection, Immunity and Inflammation, College of Medical, Veterinary and Life Sciences, University of Glasgow, Glasgow, United Kingdom; 8 Unit of Cellular and Molecular Immunology, Vrije Universiteit Brussel, Brussels, Belgium; 9 Department of Structural Biology, VIB, Vrije Universiteit Brussel, Brussels, Belgium; New York University School of Medicine, UNITED STATES

## Abstract

African trypanosomiasis is a deadly neglected disease caused by the extracellular parasite *Trypanosoma brucei*. Current therapies are characterized by high drug toxicity and increasing drug resistance mainly associated with loss-of-function mutations in the transporters involved in drug import. The introduction of new antiparasitic drugs into therapeutic use is a slow and expensive process. In contrast, specific targeting of existing drugs could represent a more rapid and cost-effective approach for neglected disease treatment, impacting through reduced systemic toxicity and circumventing resistance acquired through impaired compound uptake. We have generated nanoparticles of chitosan loaded with the trypanocidal drug pentamidine and coated by a single domain nanobody that specifically targets the surface of African trypanosomes. Once loaded into this nanocarrier, pentamidine enters trypanosomes through endocytosis instead of via classical cell surface transporters. The curative dose of pentamidine-loaded nanobody-chitosan nanoparticles was 100-fold lower than pentamidine alone in a murine model of acute African trypanosomiasis. Crucially, this new formulation displayed undiminished in vitro and in vivo activity against a trypanosome cell line resistant to pentamidine as a result of mutations in the surface transporter aquaglyceroporin 2. We conclude that this new drug delivery system increases drug efficacy and has the ability to overcome resistance to some anti-protozoal drugs.

## Author Summary

Drug resistance is complicating the treatment of parasitic diseases including African trypanosomiasis, a fatal disease if left untreated. Development of a vaccine is unlikely due to parasite antigenic variation. Current chemotherapy relies primarily on four drugs. Three of these drugs access the cell’s interior through surface transporters and resistance mechanisms are largely associated with loss-of-function mutations in the involved surface drug transporters. We reasoned that using an alternative drug entrance would circumvent parasite resistance due to mutation in a surface transporter. We have developed a drug nanocarrier that consists of polymeric nanoparticles coated with a single domain antibody that targets the trypanosome surface. This new formulation reduces the minimal curative dose and, most importantly, circumvents drug resistance in a resistant cell line as a result of mutations in the surface transporter that mediate drug uptake. This study presents a proof-of-concept of a novel technology for reversing transporter-related drug resistance with applications to other infectious diseases.

## Introduction

Human African trypanosomiasis, also known as sleeping sickness, is caused by the flagellated protozoa *T*. *b*. *gambiense* and *T*. *b*. *rhodesiense*, which are transmitted by tsetse flies of the genus *Glossina* from human and/or animal reservoirs [1–2]. Trypanosomes evade their hosts’ humoral immune response through continuous variation of the variant surface glycoprotein through a process called antigenic variation, hampering the generation of conventional vaccines [3]. Therefore, treatment of African trypanosomiasis with chemotherapy is the only viable control option. HAT chemotherapy relies primarily on four drugs: pentamidine, suramin, melarsoprol and, most recently, eflornithine/nifurtimox combination therapy (NECT) [4]. All of them have limitations, ranging from problems with poor efficacy and acute toxicity to drug resistance [5].One of the most promising new therapeutic approaches for improved chemotherapy focuses on the design of polymeric nanostructures as drug delivery systems. Chitosan is a biodegradable and biocompatible compound obtained by partial deacetylation of the natural polymer chitin. Chitosan may be prepared as nanoparticle (NP) drug carriers functionalized with agents such as polyethylene glycol (PEG). Targeted delivery of nanoparticles enhances the effectiveness of the treatment, minimizes toxicity and prevents drug metabolism and elimination [6]. Active targeting and delivery can be achieved by coupling ligands or antibodies onto the surface of the NPs. For example, the single-domain antibodies (called nanobodies) are small antibodies fragments, derived from camelids heavy chain antibodies through recombinant gene technology, with unique antigen recognition properties; they can be used to target biological structures or specific cell types [7], including African trypanosomes [8–9].

Here we have developed a new polyvalent drug delivery system for the treatment of African trypanosomiasis based on PEGylated chitosan nanoparticles coated with a nanobody that specifically recognizes conserved cryptic epitopes on the parasite surface [8]. Nanoparticles were loaded with the trypanocidal drug pentamidine and its effectiveness was assayed in vitro and in vivo against *T*. *brucei* and a pentamidine resistant cell line.

## Results and Discussion

### Generation and characterization of the drug delivery system

We designed a nanocarrier for drugs, consisting of pentamidine-loaded functionalized PEGylated-chitosan nanoparticles, coated by a single-domain antibody (nanobody) derived from camel heavy-chain antibodies, which targets the surface of *T*. *brucei* [8–9]. More precisely, this nanobody, known as NbAn33, specifically recognizes a conserved N-linked high mannose oligosaccharide present in most VSGs [8–10]. The nanobody epitope is located close the parasite surface membrane, inaccessible for large molecules such as conventional antibodies. Heterofunctional PEG chains were employed to link NbAn33 to chitosan NPs. The molecular weight of the PEG used, 3 kDa, was an important parameter in the design of the nanocarrier. As previously reported [11], its chain length (26 nm) allows the nanobody linked to the NPs to reach its recognition epitope concealed within the densely packed VSG surface coat (about 10–15 nm thick) acting as an anchor rope for the nanoparticle.

Pentamidine-loaded functionalized PEGylated-chitosan nanoparticles coated by NbAn33, NbAn33-pentamidine-chNPs, were generated by a coacervation method [12] which allowed the generation of well-stabilized spherical NPs with an average size of ~ 135 nm in diameter [13–15] (S1 Table). A Zeta (ζ) potential value analysis showed no substantial differences between the surface charge properties of pentamidine loaded NPs and empty NPs, indicating that pentamidine was trapped inside NPs rather than just absorbed at the surface (S1 Table). PEGylation of chitosan NPs was qualitatively confirmed by nuclear magnetic resonance. The maximum pentamidine concentration loaded into NPs, expressed as entrapment efficiency and drug loading capacity, was 67% and 23%, respectively. The in vitro characterization of pentamidine release showed a biphasic profile at physiological pH: 40% of the encapsulated pentamidine was rapidly released within the first 12 h, while the remaining 60% was released at a constant rate during the following ~5 days (S1 Fig). Interestingly, the NPs showed a pH-responsive drug release (S1 Fig), likely due to swelling/degradation of the NP matrix at acid pH. This behaviour may be advantageous for the intracellular delivery of pentamidine in acidic compartments. Finally, results from blood compatibility studies indicated a broad in vivo safety margin for all the nanoparticulate formulations (S2 Table).

### In vitro trypanotoxicity studies

**Fig 1:**
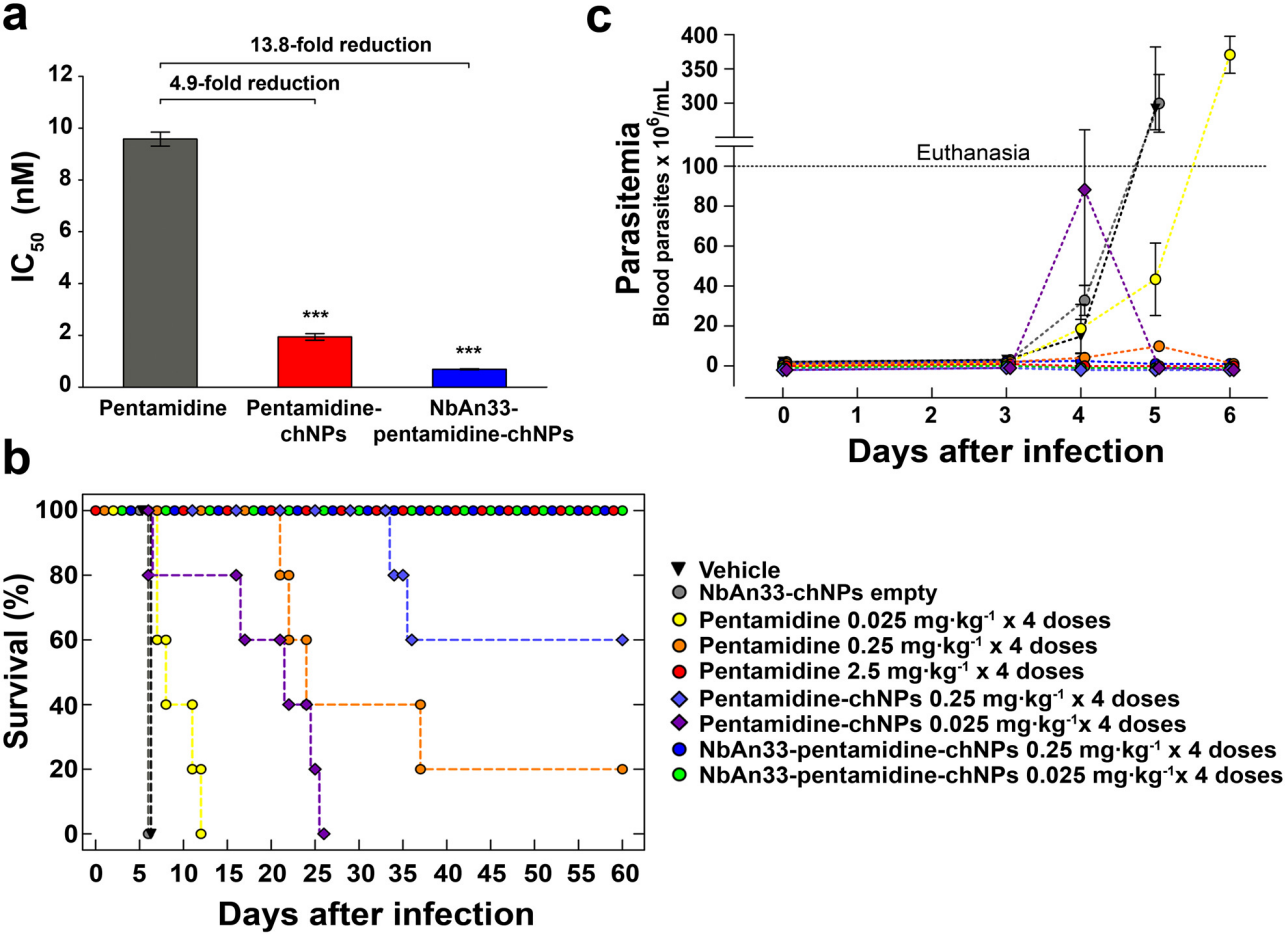
Sensitive profile of *T*. *brucei* bloodstream forms. (a) IC_50_ analysis. Pentamidine (grey column); pentamidine-loaded PEGylated chitosan nanoparticles (pentamidine-chNPs, red column) and nanobody-coated pentamidine-loaded PEGylated chitosan nanoparticles (NbAn33-pentamidine-chNPs, blue column). Errors bars indicate S.D. from 3–9 independent experiments. Statistical significance was ***, p<0.001. (b) Therapeutic effect in *T*. *brucei* acute infection mouse model. Survival (Kaplan-Meier plot) of female C57BL/6J mice infected with *T*. *brucei* AnT1.1 (1 x 10^4^ parasites). The treatment started once the parasites were detected in blood, at the 3rd day after inoculation and consisted in a daily dose in four consecutive days. Treatment with pentamidine, pentamidine-chNPs, NbAn33-pentamidine-chNPs, NbAn33-chNPs empty (nanobody-coated non pentamidine-loaded PEGylated chitosan nanoparticles) and vehicle (physiological saline solution). (c) Parasitemia in *T*. *brucei* acute infection mouse model. Treatment with vehicle (physiological saline solution), NbAn33-chNPs empty (nanobody-coated PEGylated-chitosan nanoparticles), free pentamidine, pentamidine-chNPs (pentamidine-loaded PEGylated chitosan nanoparticles), NbAn33-pentamidine-chNPs (nanobody-coated pentamidine-loaded PEGylated chitosan nanoparticles).

The-inhibitory concentration (IC_50_) value of free pentamidine for bloodstream trypanosomes was 9.6 ± 0.3 nM (Fig 1A). The trypanolytic effect of pentamidine was significantly improved when it was loaded into NbAn33-pentamidine-chNPs. The IC_50_ value was 0.69 nM, which represents an approximately 14-fold reduction in drug concentration relative to free pentamidine (P<0.0001) (Fig 1A). To evaluate the contribution of NbAn33 to nanoparticles efficacy, the effect of pentamidine-loaded PEGylated chitosan nanoparticles (pentamidine-chNPs) that had not been coated by the NbAn33 was tested in parallel. The trypanocidal activity of non-coated NPs was still higher than free pentamidine (IC_50_ value of 1.94 nM; P<0.0001, versus free pentamidine) but lower than NbAn33-pentamidine-chNPs (P<0.0001) (Fig 1A). One factor in the relative effectiveness of the uncoated NPs compared to free pentamidine may be the electrostatic interactions between positively charged nanoparticles with the slightly negatively charged surface of the bloodstream forms. As expected, neither unloaded PEGylated chitosan nanoparticles (chNPs-empty) nor empty nanobody-coated PEGylated-chitosan nanoparticles (NbAn33-chNPs) had any effect on parasite viability (S2 Fig).

### In vivo therapeutic studies

The minimal full curative dose of pentamidine in a mouse model of acute infection of *T*. *brucei* was previously established as four doses of 2.5 mg·kg^-1^ administrated daily by intraperitoneal injection in four consecutive days, starting upon detection of parasites in blood (day 3 after infection) [16–17] and this was also curative in our model of infection (Fig 1B). Mice were intraperitoneally infected with 10^4^ parasites. In the group of mice treated with a 10-fold lower dose of free pentamidine (4×0.25 mg·kg^-1^) the parasites disappeared from the peripheral blood after the third dose (Fig 1C). However, the infection relapsed and the mice began to die at day 22 after infection, curing only 20% of the treated animals (Fig 1B).

Having established the suboptimal pentamidine curative dose (4×0.25 mg·kg^-1^), we treated mice with an equal dose of pentamidine loaded into NbAn33-chNPs. Clearance of parasites from this group was complete after the first dose and the treatment successfully cured 100% of the animals (Fig 1B and 1C). Infected mice were also treated with the same pentamidine dose loaded into chNPs that were not coated with the nanobody. In this group parasites disappeared from the blood after the first dose, however 40% of the treated mice succumbed to the infection (Fig 1B and 1C). Next, a dose that was 100-fold lower than the minimal curative dose of pentamidine was tested. At that low dosage (4×0.025 mg·kg^-1^), free pentamidine did not cure any of the mice from trypanosome infection. Although the parasitemia levels increased less rapidly than in vehicle treated mice the parasites never disappeared from the blood and all mice died after 7–12 days of infection (Fig 1B and 1C). As observed in vitro, pentamidine loaded into non coated NPs was more effective than free pentamidine probably as a consequence of a sustainable drug release from the nanoparticles. Remarkably, treatment with 4 doses of NbAn33-pentamidine-chNPs at 0.025 mg·kg^-1^ was able to eliminate the parasitemia after the second dose, curing all treated mice (Fig 1B and 1C). However, the same low dose of pentamidine loaded into chNPs non-coated by the NbAn33, despite clearing the parasites from the blood after the third dose, did not cure mice, with 100% of the treated animals dying from the infection (median survival time 22 days).

**Fig 2:**
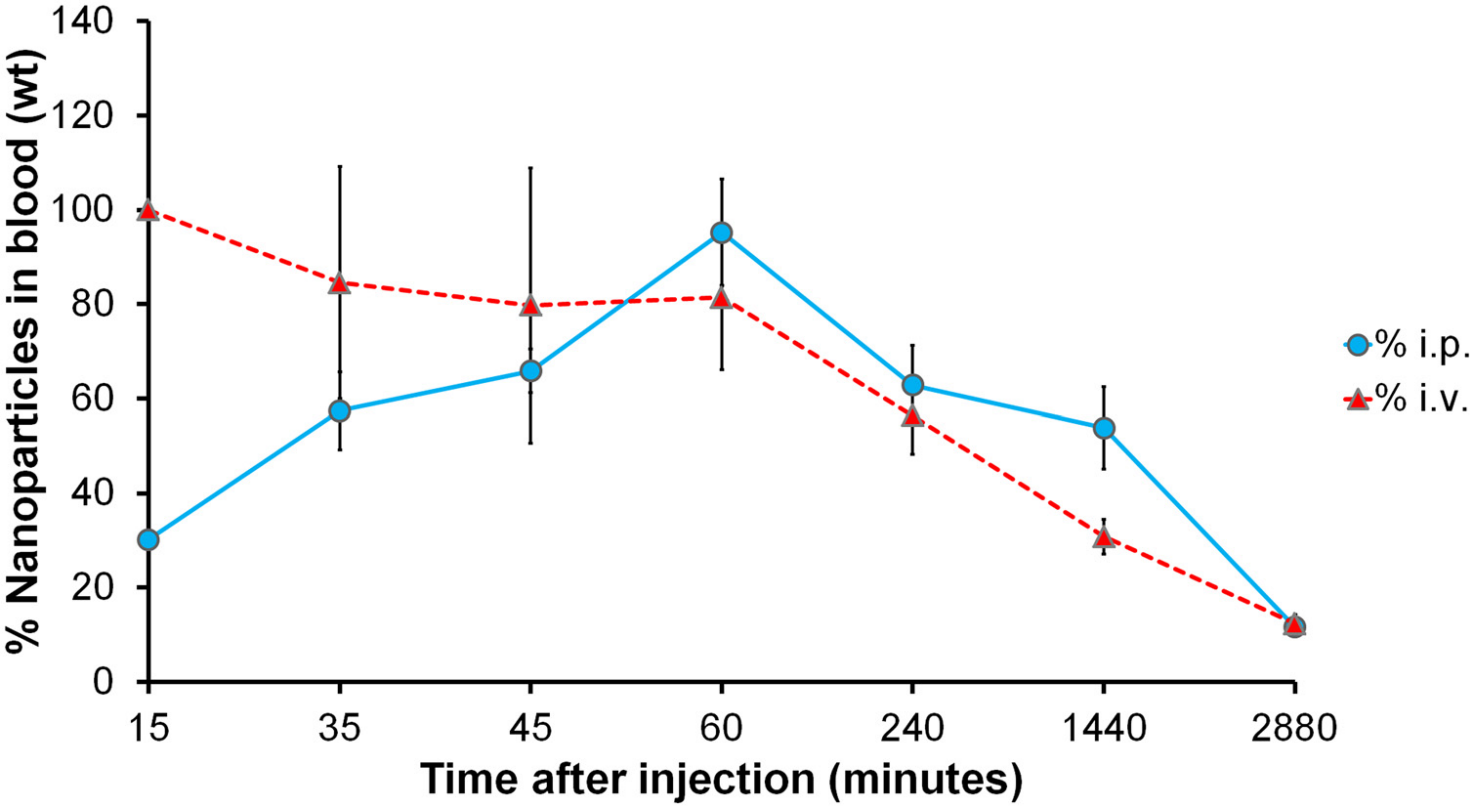
Circulation kinetics of fluorescent chitosan nanoparticles in mouse model. Percentage of the initial dose in peripheral blood when injected via intravenous (i.v.) and intraperitoneal (i.p.) vs. time. Error bars represent the S.D. from 5 mice.

In order to investigate the circulation kinetics of NbAn33-chNPs injected intraperitoneally, we compared the percentage of the dose of NbAn33-chNPs in peripheral blood when administered intraperitoneally versus intravenously. Thus, NbAn33-chNPs labelled with the infrared fluorophore (DY-649) were administered intravenously or intraperitoneally and fluorescence in blood was measured at various time points. Nanoparticles concentration in blood at 15 min after intravenous injection was taken as reference value (100%). About 90% of the NbAn33-chNPs intraperitoneally administered was detectable in blood after 60 min post injection (Fig 2). These results demonstrated that the intraperitoneal route was suitable for NbAn33-chNPs administration.

### Uptake of NbAn33-coated NPs in bloodstream trypanosomes

**Fig 3:**
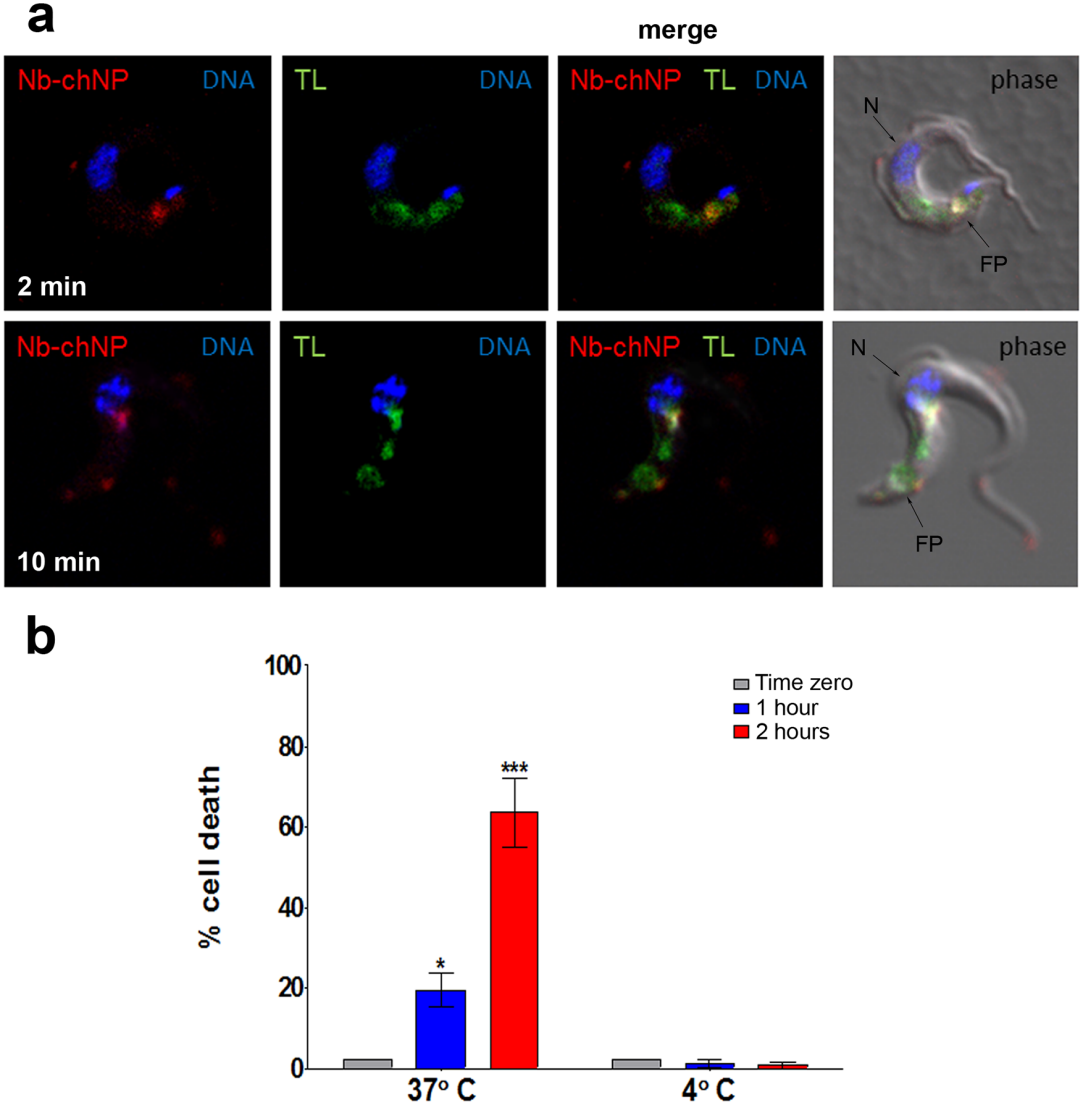
Endocytosis of NbAn33-chNPs. (a) Bloodstream trypanosomes observed by fluorescence microscopy after incubation with NbAn33-chNPs-Alexa Fluor 594 (red) and tomato lectin-FITC (TL, green) as described in Materials and Methods. Samples were taken after 2 minutes (bottom panel) and 10 minutes (top panel) of incubation. DNA is stained with DAPI (blue). Regions of colocalization appear yellow in merged images. (b) Parasite viability after incubation with NbAn33-pentamidine-chNPs at 37° and 4°C for 2 h. Cell death was estimated by propidium iodide staining and FACS analysis at three time points. Error bars represent the S.D. from three independent experiments. Statistical significance was *, p<0.05; ***, p<0.001.

Three previous studies have shown that NbAn33 is internalized by endocytosis in bloodstream trypanosomes [18–20]. Therefore, one would anticipate that nanoparticles coated by the nanobody NbAn33 would enter the cell via this route. Nanoparticles uptake was monitored by fluorescence after incubating bloodstream trypanosomes with Alexa-labelled-NbAn33-chNPs at 37°C. As expected, the fluorescence signal concentrates rapidly in the flagellar pocket (FP) (Fig 3A) and after in the endocytic pathway colocalizing with fluorescent tomato lectin, a marker of this route in bloodstream African trypanosomes [21] (Fig 3A). Moreover, a comparative study of the trypanocidal actvity of NbAn33-pentamidine-chNPs at 4°C and 37°C showed that at 37°C trypanotoxicity was time dependent (20% and 65% of cell death after 2 and 4 hour of incubation in the presence of pentamidine (30 μM) (Fig 3B). However, no activity was observed at 4°C under the conditions of the experiment. Together, these results indicate that NbAn33-chNPs internalization depends on the endocytic process.

### Effectiveness of pentamidine loaded NbAn33-chNPs in a pentamidine resistant cell line

**Fig 4:**
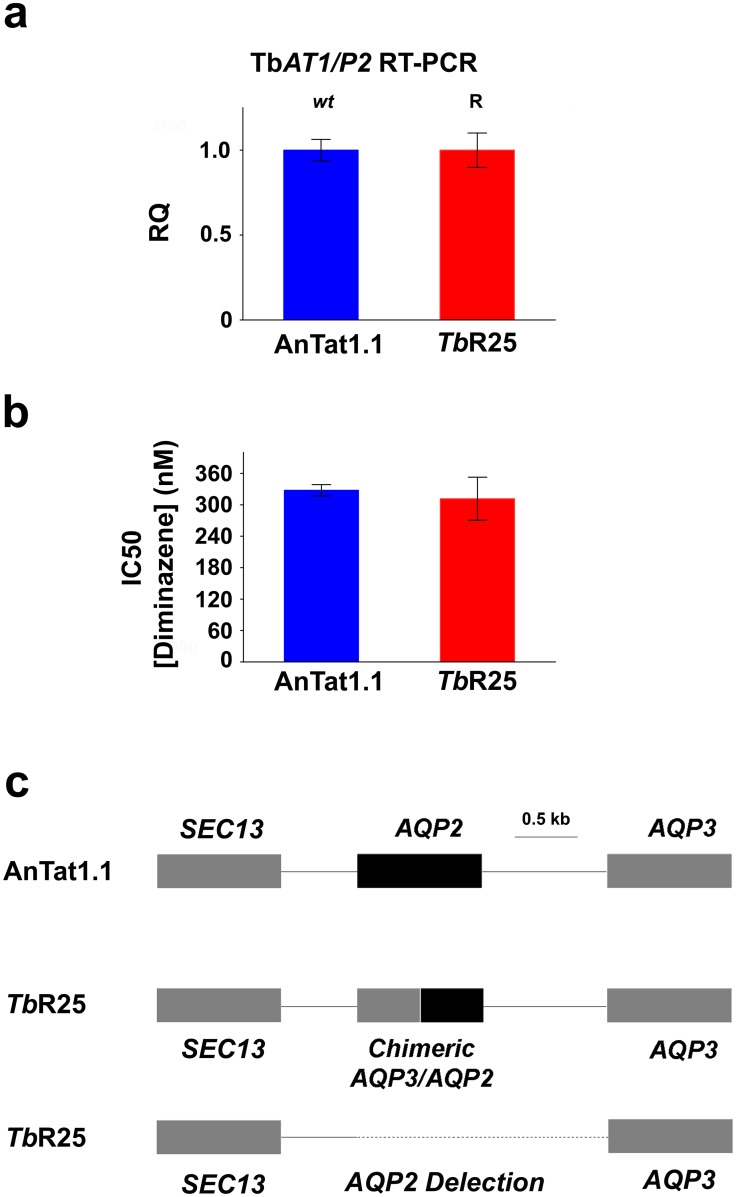
Characterization of the pentamidine resistant strain *Tb*R25. (a) Relative quantification (RQ) of *TbAT1/P2* expression in wild type AnTat 1.1 and *Tb*R25 strains estimated by qRT-PCR. (b) IC_50_ value for diminazene aceturate in the same strains. Error bars indicate S.D. from 3 replicates. (c) Schematic illustration of the *AQP2/AQP3* locus showing the heterozygote character of *Tb*R25 strain with a chimeric gene in one allele and the complete deletion of *AQP2* gene and the intergenic region in the other (deletion from position 3441867 to 3443663 in chromosome 10, -Tb927_10_v5).

Resistance to pentamidine in *T*. *brucei* is associated with mutations in cell surface transport proteins, specifically in the TbAT1/P2-adenosine transporter [22–24] and in the aquaglyceroporin 2 (AQP2) channel [25–28]. However, pentamidine loaded into nanoparticles coated by the nanobody NbAn33 is internalized by the endocytic route (Fig 3). We reasoned that this alternative drug entrance would circumvent parasite resistance to the drug. To test the hypothesis that the trypanocidal action of encapsulated pentamidine was not dependent on cell surface transporters, a *T*. *brucei* pentamidine-resistant cell line was selected after in vitro exposure to increasing concentrations of the drug (up to 50 nM). The resistant clonal cell line, designated *Tb*R25, was genetically and functionally characterized to determine whether resistance was due to a mutation in one of the known pentamidine transporters in the plasma membrane. The *TbAT1/P2*-adenosine transporter gene was amplified from wild type and *Tb*R25 genomic DNA. No differences in length or sequence were observed between both PCR products. Moreover, no significant change in *TbAT1/P2* expression levels was noted by real-time qRT-PCR analysis between wild type and resistant cell line (Fig 4A). Finally, *Tb*R25 and wild type trypanosomes were equally sensitive to diminazene aceturate (Fig 4B), another trypanocidal aromatic diamidine where uptake is almost exclusively mediated by the TbAT1/P2 adenosine transporter [29–31] Together, these data demonstrated that the adenosine transporter was not involved in the resistance to pentamidine in the *Tb*R25 cell line.

We next searched for mutations in the genomic locus encoding the closely related AQP2 and AQP3 aquaglyceroporins. Interestingly, genomic PCR analysis of the *AQP2*/*AQP3* locus showed the deletion of *AQP2* gene in one allele and an *AQP3/AQP2* chimeric gene in the other allele (Fig 4C and S3 Fig). In the chimera *AQP3/AQP2* the first 453 nucleotides were from *AQP3* and the remaining 462 nucleotides, from *AQP2*. In contrast, the *AQP3* sequence was intact in both alleles. Rearrangements of the *AQP2/AQP3* locus resulting in the loss of the wild-type *AQP2* gene loss also occur in the field, where this genotype is associated with melarsoprol treatment failure [27]. This result was in agreement with the previous observation that loss of *AQP2* renders *T*. *brucei* much less sensitive to pentamidine [25, 27–28]. Our results thus suggest that the mechanism of acquired resistance to pentamidine in *Tb*R25 cell line was due to the deletion of *AQP2* in one allele and the presence of the chimera *AQP3/AQP2* in the other allele (Fig 4D and S3 Fig). Although a number of chimeric rearrangements in the AQP2/AQP3 have been reported [27–28] this is the first rearrangement where it is the amino terminal end is of AQP3 and the carboxy-terminal sequence is from AQP2 rather than the other way around. Recently, it has been published a model of pentamidine permeation through TbAQP2 [32], proposing that three bulky amino acids, present in the TbAQP3 pore but not in the TbAQP2 pore, help explain the difference in pentamidine permeation between these otherwise closely-related proteins. According to this model, the *Tb*R25 rearrangement introduces at least one bulky amino acid, tryptophan 102 of AQP3, into the pore, restricting the passage of the relatively large pentamidine molecule.

**Fig 5:**
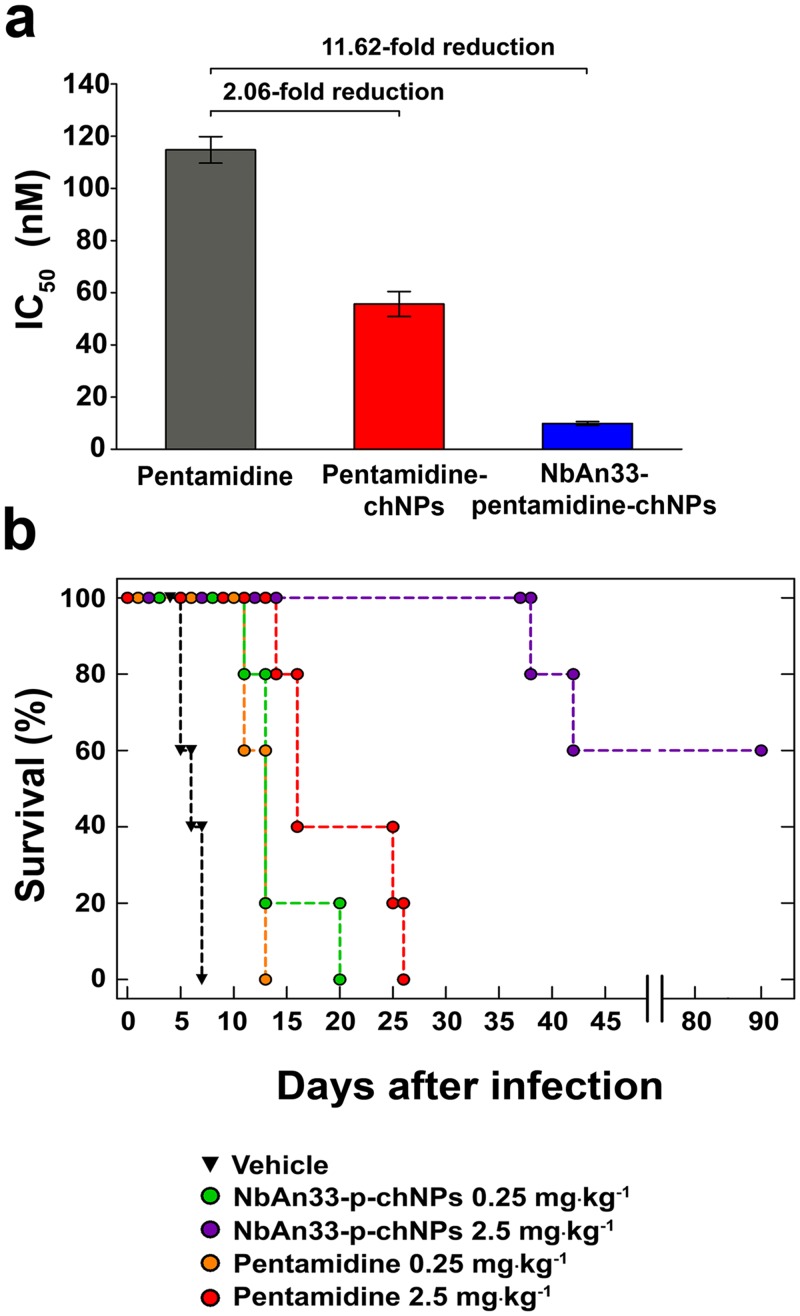
Sensitive profile of the pentamidine resistant strain *Tb*R25. (a) IC_50_ analysis. Free pentamidine (grey column); pentamidine-chNPs (red column) and NbAn33-pentamidine-chNPs (blue column). Errors bars indicate SEM from 3–9 independent experiments. Fold reductions are indicated in the graph. (b) Therapeutic effect in *Tb*R25 acute infection mouse model. Survival (Kaplan-Meier plot) of female C57BL/6J mice infected with *Tb*R25 (inoculum 2.5 x 10^6^ parasites).

Having established that the resistance mechanism was due to the absence of the wild-type coding sequence of *TbAQP2*, we decided to test whether NbAn33-pentamidine-chNPs were able to circumvent this resistant mechanism by avoiding the classical uptake through transporters. The IC_50_ value for pentamidine in the pentamidine-resistant strain *Tb*R25 was 115 ± 5 nM (Fig 5A). However, NbAn33-pentamidine-chNPs reduced the IC_50_ to 9.9 ± 0.7 nM, similar to the IC_50_ of the wild type strain to free pentamidine (9.6 ± 0.3 nM). Uncoated pentamidine-chNPs were also able to reduce the IC_50_ of *Tb*R25, although only 2.1-fold relative to free pentamidine (P<0.001) (Figs 1A and 5A).

Next, we tested whether the new delivery system was able to overcome pentamidine resistance in vivo. As observed for other pentamidine resistant trypanosomes [24, 26], the *Tb*R25 cell line was significantly less virulent than the wild type. In order to establish infection the mice were subjected to immunosuppression and inoculated with a parasite load 250-fold higher than required with our standard strain AnTat 1.1. Once infected, the animals were treated with the curative dose for wild type trypanosomes (four daily doses of 2.5 mg·kg^-1^) of free pentamidine and its equivalent loaded within the chNPS coated with NbAn33. All mice treated with free pentamidine died after infection, whereas 60% of the mice survived following treatment with NbAn33-pentamidine-chNPs, and the mice that did succumb to the infection lived substantially longer than those treated with free pentamidine (Fig 5B). The difference observed in NbAn33-pentamidine-chNPs effectiveness in *Tb*R25 resistant cell line compared to wild type might be related to the loss of virulence and the fact that mice were immunosuppressed before *Tb*R25 infection with 250 times higher parasite load. The cause of this loss of virulence is not known but is independent of mutations in the *AQP2* genes since such mutations are present in field isolates [27]. Therefore, the virulence attenuation phenotype of *Tb*R25 is likely to be associated to laboratory-selected *T*. *brucei* mutants.

For the continued treatability of African trypanosomiasis, as for many other infectious diseases, the emergence and spread of drug resistance is the major concern together with the absence of vaccines. Most of trypanocidal drugs do not diffuse freely across parasite cell membrane, but several transporters are responsible for their uptake, thereby making a crucial contribution to their selectivity [33–34]. In African trypanosomes, resistance mechanisms have generally been associated with mutations that reduce drug import, through loss of function or changes in substrate selectivity. Notably, there is currently little evidence for the involvement of drug export transporters such as multi-drug resistance-associated carriers in acquired drug resistance, as observed for other protozoa such as *Plasmodium* and *Leishmania spp* [35–36]. A recent genomic-scale screening has linked most of the current HAT drugs to specific genes encoding surface proteins involved in their uptake [22], confirming some previous results. Eflornithine resistance was associated with loss of amino acid transporter family member AAT6 [22, 37–38], suramin was linked to invariant surface glycoprotein ISG75, melarsoprol to adenosine transporter TbAT1/P2 [38–41], pentamidine to P-type H+-ATPases that maintain the proton-motive force across the plasma membrane, and pentamidine/melarsoprol cross-resistance to aquaglyceroporins, specifically to aquaglyceroporin 2, which encodes the High Affinity Pentamidine Transporter (HAPT1) and controls the susceptibility to both drugs [25, 27–28].

The use of chitosan nanoparticles-based therapy allows drug release to be tailored to the specific target site through the choice of various polymer and copolymer combinations and formulation procedures [42–43]. When the nanocarrier was designed, all possible parameters that could influence the grade of success of our nanodevice were taken into account. One of the most important was the pathway that the nanocarrier has to follow to deliver the drug. As shown in Fig 3A, once the nanoparticles reach the surface of the parasite they are taken up by endocytosis in the flagellar pocket. Along the endocytic pathway the pH is decreasing, reaching the lowest value (~pH 5) in the lysosome. Previous reports comparing different polymeric NPs have concluded that drug release from chitosan NPs is pH dependent [44], making chitosan a suitable polymer for this specific nanocarrier.

Pentamidine-loaded poly (D,L-lactide) and polymethacrylate nanoparticles have been previously used against *Leishmania* (*L*. *infantum and L*. *major*) [45–47]. Pentamidine-loaded nanoparticles were between 3.3 and 6 times more effective than free pentamidine against *Leishmania* in a murine model vivo. By contrast, NbAn33-pentamidine-chNPs were 100 times more effective than free pentamidine against *T*. *brucei* in vivo. However, these results are not directly comparable because *Leishmania* is an intracellular parasite while *T*. *brucei* is extracellular. Another crucial difference is the use of the nanobody NbAn33 for active targeting which notably increases the effectiveness of the formulation as shown in this study.

In summary, the development of chitosan nanoparticles loaded with current trypanocidal drugs coated by a specific nanobody against trypanosomes can reduce the minimal curative dose of these drugs, enhancing their efficacy, minimizing the toxicity and circumventing resistance mechanisms associated to mutation in surface transporters. The significance of the report is not limited to a single nanobody used to demonstrate the technology, nor to a single drug, nor indeed to trypanosomiasis. Due to its versatility, the possibilities that offer this targeted nanobody-system are enormous as it can be adapted to encapsulate any substance with a reported biological action. This opens up a plethora of new possible therapies to treat different infectious diseases. The most urgent issue in the treatment of infectious disease today is that resistance to drugs is spreading much faster than new drugs are being developed and approved. The use of encapsulated, nanobody-targeted drugs as described here has the potential to reverse resistance to many first-line treatments.

## Materials and Methods

### Ethics statement

All experiments complied with the with the guidelines of the European Convention for the Protection of Vertebrate Animals used for Experimental and other Scientific Purposes (CETS n° 123) and were approved by the Ethics Committee of the Spanish National Research Council (CSIC, file UEA2011/JAGS/1).

### Parasites

Bloodstream forms of monomorphic *T*. *brucei* AnTat 1.1 strain (Institute of Tropical Medicine, Belgium) were grown in axenic culture at 37°C and 5% CO_2_ in HMI-9 medium supplemented with 20% heat-inactivated foetal bovine serum (Gibco) [48].

### Nanobody NbAn33

The nanobody NbAn33 used in this study was selected as previously described [8]. NbAn33 recognizes a glycosylated (Man9–5GlcNAc2) epitope on *T*. *brucei* VSGs, as indicated by its binding to synthetic Man9 and Man7 and by its competition for binding with concanavalin A [8]. Accordingly, NbAn33 binds equally well to several VSGs, such as the MiTat 1.1, MiTat 1.4, MiTat 1.5 and ETat 1.2 VSGs, which represent different VSG classes and share the conserved N-linked Man5–9 carbohydrate [8–9].

### Preparation of nanobody-coated pentamidine-loaded functionalized PEGylated-chitosan nanoparticles

For the synthesis of copolymer chitosan-graft-PEG (chitosan-g-PEG), chitosan hydrochloride (80 mg) was dissolved in 11.5 mL of filtered deionized water. To which MeO-PEG-CH_2_CO_2_H (14.2 mg) and N-hydroxysuccinimide (NHS, 1.6 mg) were added. 1-ethyl-3-(3-dimethylaminopropyl) carbodiimide (EDC, 21.7 mg) was then added gradually and the resulting solution was stirred at room temperature for 22 h. The solution was ultrafiltered (0.22 μm pore size) and lyophilized.

PEGylated chitosan nanoparticles were prepared by a coacervation method avoiding the use of toxic organic solvents [13–15]. Briefly, the copolymer chitosan-g-PEG (1%, w/v) was dissolved in 10 mL of an aqueous solution of acetic acid (2%, v/v). Next, pluronic F-68 and pentamidine (isethionate salt, Sigma) were added at increasing concentrations up to 2% (w/v) and 0.01 M, respectively. Approximately 2.5 mL of a solution of sodium sulphate (20%, w/v) was added drop wise (0.5 mL·min^-1^) to the chitosan solution under mechanical stirring (2,000 rpm). The stirring was continued for 1 h to ensure the formation of pentamidine loaded functionalized PEGylated-chitosan nanoparticles (pentamidine-chNPs). The newly formed pentamidine-chNPs were cleaned by 3 consecutive cycles of centrifugation for 30 min at 11,000 rpm in a Centrikon T-124 high-speed centrifuge (Kontron) and re-dispersion in double-distilled water until the conductivity of the supernatant was ≤ 10 μS·cm^-1^, measured in a Crison micro pH 2001 conductometer (Crison). Finally, nanobody NbAn33at 1 mg·mL^-1^ was added to the pentamidine-NPs suspension (10:1 weight ratio) in 10 mL of phosphate buffered saline (PBS, pH 7.4) containing EDC and NHS. The reaction was left for 3 h at 25°C under mechanical stirring (200 rpm). The resulting NbAn33-coated pentamidine-chNPs (NbAn33-pentamidine-chNPs) were submitted to a single wash cycle by centrifugation for 30 min at 11,000 rpm in a Centrikon T-124 high-speed centrifuge (Kontron) and suspended in 10 mL of physiological saline solution (0.09%w/v NaCl). Non-pentamidine loaded nanoparticles (NbAn33-chNPs) were prepared in parallel.

### Nanoparticles characterization

Mean particle diameter (± standard deviation, S.D.) was determined by photon correlation spectroscopy (PCS) using a Malvern 4700 analyzer (Malvern). The scattering angle was set at 60° and the measurement was made after suitable dilution of the aqueous nanoparticle suspensions (0.1%, w/v). The stability of the formulations was evaluated by measuring the size of the particles after 1 month of storage at 4°C in water.

The electrophoretic mobility measurements can qualitatively distinguish the mode of pentamidine association with the NPs: encapsulation within the NP matrix or adsorption on the NP surface. Briefly, the measurements were performed in 0.1% (w/v) aqueous suspensions of NbAn33-chNPs and NbAn33-pentamidine-chNPs in 1 mM KNO_3_ pH 6 using a Malvern Zetasizer 2000 electrophoresis device (Malvern). Measurements were performed after 24 h of contact of NPs in water under mechanical stirring (50 rpm) at 25°C. The experimental uncertainty of the measurements was below 5%. The electrophoretic mobility was converted into zeta potential (ζ mV) values as described by O’Brien and White [13].

### Quantification of pentamidine loaded into the chitosan nanoparticles

Ultraviolet-visible spectrophotometry (UV—Vis) was used to quantify the amount of pentamidine loaded into NPs at a wavelength of 261 nm in a 8500 UV-Vis Dinko spectrophotometer (Dinko). The spectrophotometric method employed was validated by ultra-high-pressure liquid chromatography and mass spectrometry (UPLC-MS) in an Acquity UPLC/QTOF Synapt G2 (Waters). After NbAn33-pentamidine-chNPs synthesis, the supernatant was obtained by double centrifugation for 30 min at 11,000 rpm and 25°C in centrifuge machine Centrikon T-124 high-speed centrifuge (Kontron) and the amount of pentamidine was measured in triplicate. Drug incorporation to NPs was expressed in terms of pentamidine entrapment efficiency (%) (encapsulated drug [mg]/total drug in the NP suspension [mg] × 100) and pentamidine loading (%) (encapsulated drug [mg]/carrier [mg] × 100).

### In vitro release studies of pentamidine from chitosan nanoparticles

The study of pentamidine release was performed using the NbAn33-pentamidine-NPs with the maximal loading and entrapment efficiency reached (~23% and ~ 66%, respectively, S1 Table). The assay was performed at 37°C in triplicate following the dialysis bag method using Spectra/Por 6 dialysis membrane tubing (Spectrumlabs). The release medium for neutral and acidic conditions was PBS at pH 7.4 and pH 5 respectively. The dialysis bag with pore size 2000 Da retained the NPs, but allowed the free pentamidine to diffuse through the membrane into the release medium. Briefly, 1 mL of nanoparticle suspension (containing 0.35 mg·mL^-1^ of pentamidine) was pipetted into the bags, and the two ends fixed by clamps. The bags were then placed in a conical flask filled with 100 mL of the receiving phase, and were stirred at 200 rpm. Samples of 1 mL were taken at different times (0.5 h, 1 h, 3 h, 6 h, 9 h and 24 h, and 2, 3, 4 and 5 days) and analyzed for pentamidine content by UV—Vis spectrometry at 261 nm. To maintain dialysis conditions an equal volume of the release medium at 37°C was added after taking each sample.

### Blood compatibility and cytotoxicity

The interaction of control (pentamidine unloaded) PEGylated chitosan nanoparticles, NbAn33-chNPs, pentamidine-chNPs, and NbAn33-pentamidine-chNPs, with blood components was investigated in triplicate following a well-defined procedure [49]. The in vitro effect of the nanoparticulate formulations on erythrocyte lysis was evaluated using PBS as negative control (0% lysis) and the non-ionic surfactant Triton X-100 (1%, w/v) as positive control (100% lysis) of hemolysis. Briefly, EDTA-anticoagulated blood was centrifuged at 1,500 rpm for 20 min to remove the plasma fraction. The original volume was then replaced with 150 mM NaCl. Upon repetition of this step, the final suspension was diluted 1:10 with 100 mM PBS. About 100 red blood cells·mL^-1^ were incubated at 37°C for 2 h with the nanoparticles (0.05%, w/v) under mechanical stirring (200 rpm), and centrifuged at 1,500 rpm for 20 min. Hemoglobin release was evaluated by measuring the UV absorbance of the supernatant at 545 nm. Hemolysis (%) was determined as ([absorbance of test sample—absorbance of control] / highest absorbance of positive control) × 100.

Interaction of platelets with nanoparticulate systems may determine their activation (and aggregation) which generates thrombotic complications and blood incompatibilities. This process is quantified by enzyme-linked immunosorbent assay (ELISA) in terms of soluble P-selectin (sP-selectin) release after incubating the nanoparticles with blood. Blood samples were centrifuged at 1,000 rpm for 20 min to remove platelet-rich supernatant. Then, blood was again centrifuged and mixed with previously extracted plasma to obtain platelet-rich plasma (PRP). Platelet-poor plasma (PPP) was obtained by centrifuging the remaining blood at 3,000 rpm for10 min. The PRP was then diluted 1:100 with 1% ammonium oxalate, and adjusted to a final platelet concentration of 1·10^8^ mL^-1^. A sample of 0.1 mL of PRP was incubated for 1 h with 50 mg of nanoparticles at 37°C. The supernatant was then centrifuged 10 min at 5,000 rpm. Soluble P-selectin concentration in the plasma was determined using ELISA kit (eBioscence,) following to the manufacturer’s indications.

Opsonization of nanoparticles with complement components (i.e., C3a and C5a) leads to blood clearance by the reticuloendothelial system (RES). Complement activation was quantified by measuring C3a release upon incubation of the nanoparticles with pooled citrated plasma obtained by blood centrifugation. C3 cleavage was monitored by measuring the formation of its activated peptide (C3a desArg) with Complement C3a des Arg Human ELISA kit (Enzo).

Plasma clotting time was determined following the Howell’s method that evaluates plasma recalcification time (PRT). T_1/2_ max was quantified as the time at which half the saturate (maximum) absorbance value was reached. PBS was used as negative control. Each blood sample was centrifuged at 3,000 rpm, 25 min at 8°C to obtain the PPP. Samples of 0.1 mL of PPP were incubated with 50 μg of nanoparticles in PBS for 5 min at 37°C in a 96 well plate. Finally, 0.1 mL of 25 mM CaCl_2_ solution was added and the plasma solution was monitored for clotting by manually dipping a silicone-coated stainless-steel wire hook into the solution to detect fibrin threads. Clotting times were estimated as the time at which the first fibrin strand was formed on the hook.

### Trypanotoxicity assay

Trypanotoxicity was determined using an adapted version of the resazurin sodium salt method [50]. Briefly, exponentially growing parasites (monomorphic *T*. *brucei* AnTat 1.1 and pentamidine resistant clonal cell line *Tb*R25) were harvested and prepared at an initial density of 2·10^5^ trypanosomes·mL^-1^. 50 μL of this trypanosome suspension was added to each well of a flat-bottom 96-well plate containing doubling dilutions of the drugs (50 μL), excepting for two rows which received only media. Eleven dilution points were tested, ranging from 680 nM to 165 pM in final pentamidine concentration. In the case of diminazene aceturate the range was between 2 μM and 2 nM. Cultured plates were incubated at 37°C in an atmosphere of 5% CO_2_ for 20 h before the addition of 20 μL of the colorimetric viability indicator resazurin sodium salt (Sigma) at 0.5 mM. After a further 4 h of incubation, the reaction was stopped by the addition of 50 μL of 3% SDS in water. The plates were read on a Tecan Infinite F200 reader (Tecan Austria GmbH) using an excitation wavelength of 535 nm and an emission wavelength of 590 nm. The experiment was performed with six replicates per concentration and repeated on at least four independent occasions. The results of fluorescence were normalized to 100% of the no-drug control. The IC_50_ was defined as the concentration of drug required to reduce the fluorescence output by 50% and its value was determined by plotting to an equation for a sigmoid curve with a variable slope, of log (test compound concentration) versus the normalized fluorescence, using Prism 5 (GraphPad Software). Statistical significance was determined by unpaired student’s t-test.

For temperature dependent trypanotoxicity assays parasites growing in logarithmic phase (5×10^5^ cell·mL^-1^) were treated with NbAn33-pentamidine-chNPs (30 μM of final pentamidine) at 37°C and 4°C respectively. Samples (5×10^5^ cells) taken at three different set time points were incubated on ice for at least 5 min, washed with phosphate-buffered saline (PBS) and incubated with propidium iodide (PI) staining solution (PBS containing 40 μg·mL^-1^ PI and 100 μg·mL^-1^ ribonuclease A) for at least 20 min on ice. The experiment was performed in parallel with untreated parasites as a control. The analysis was performed with a BD FACScanto II flow cytometer (BD Biosciences) and FlowJo software. The experiment was performed in triplicate and repeated at least three times.

### In vivo therapy experiments

Animal experimental protocols were approved by the Ethics Committee of the Spanish Council of Scientific Research (CSIC). The drug delivery system NbAn33-pentamidine-chNPs was tested in vivo against the monomorphic AnTat1.1 strain using a modification of the approach previously described [16–17]. Briefly, five female C57BL/6J mice (8-week-old; Jackson Laboratories) per group were intraperitoneally infected with 10^4^ parasites each. Once parasites were detected in the blood, at day 3 after infection, the mice were treated daily on four consecutive days with i.p. injections of pentamidine in physiological saline solution. The dosages used for each group were pentamidine 2.5 mg·kg^-1^; pentamidine 0.25 mg·kg^-1^; pentamidine 0.025 mg·kg^-1^; NbAn33-pentamidine-chNPs 0.25 mg·kg^-1^; NbAn33-pentamidine-chNPs 0.025 mg·kg^-1^; pentamidine-chNPs 0.25 mg·kg^-1^ and pentamidine-chNPs 0.025 mg·kg^-1^ (drug concentrations mg·kg^-1^ were calculated considering the molecular weight of the isethionate salt). Two control mice groups were either left untreated (injected with the same volume of physiological saline solution) or received pentamidine-free NbAn33-chNPs. We followed the parasitemia by counting the number of trypanosomes in tail-vein blood with an optical microscope with a Neubauer chamber every day during the first week, and afterwards, once per week until 60 days post-infection. Parasite survival was monitored and recorded every day until 60 days post-infection. Mice were considered cured when there was no parasitemia relapse detected in the 60 days period. Animals were humanely sacrificed when they showed severe clinical signs or when the parasitemia reached 10^8^ parasites·mL^-1^.

### Circulation kinetics of nanoparticles

Fluorescent NbAn33-chNPs were generated as previously described [51] with some modifications. Briefly, 0.5 mL of NbAn33-chNPs (1 mg·mL^-1^) were fluorescently labeled with 0.18 mM (0.2 mg) of Dy649-NHS (NbAn33-chNPs-Dy649) (Dyomics GmbH) in phosphate buffered saline (PBS) at RT for 45 min. Labeled NP were washed three times by centrifugation at 14,000 g for 30 min and resuspended in PBS at a final concentration of 1 mg·mL^-1^.

Mice were administered with 0.1 mg of NbAn33-chNPs-Dy649 (1 mg·mL^-1^) intravenously and intraperitoneally. At various time points (15 min, 30 min, 45 min, 60 min, 4 h, 24 h and 48 h), 5 μL of blood was collected from the tail and immediately diluted in 200 μL Hanks’ Balanced Salt solution (pH 7.4). The fluorescence of blood samples was measured in a plate reader (excitation 655 nm, emission 676 nm) and correlated with a standard curve of NbAn33-chNPs-Dy649 in whole blood.

### In vitro generation of *T*. *brucei* resistant cell line

*T*. *brucei* AnTat 1.1 bloodstream forms (5×10^4^ cells·mL^-1^) were growth in a 24-well plate in the presence of increasing concentrations of pentamidine, ranging from 1 nM to 10 nM. The highest concentration of pentamidine at which growth was detected was 1 nM. Parasites growing at this concentration were diluted to 5×10^4^ cells·mL^-1^ and sub-cultured into two new wells with fresh medium containing one and half and double the drug concentration, respectively. When cells were growing at 50 nM at a rate comparable to the original (wild type) strain they were diluted to an average density of 0.3 cells·mL^-1^ and cultured in a 24 wells plate. Ten clones grew and five were selected for further DNA and RNA content analysis.

### In vivo therapy experiments in a pentamidine resistant cell line

Mice were first immunosuppressed with 200 mg·kg^-1^ of cyclophosphamide (Sigma) and infected the next day with 2.5×10^6^ parasites of *Tb*R25. After ten passages in mice to adapt the resistant cell line to the host, 25 female C57BL/6J mice (6-week-old; Jackson Laboratories) were intraperitoneally injected with cyclophosphamide and infected with 2.5×10^6^ parasites the next day. Once parasites were detected in blood samples, at day 1 post infection, mice were treated daily on four consecutive days. The dosages used for each group were pentamidine 2.5 mg·kg^-1^; pentamidine 0.25 mg·kg^-1^; NbAn33-pentamidine-chNPs 2.5 mg·kg^-1^ and NbAn33-pentamidine-chNPs 0.25 mg·kg^-1^. Control mice were left untreated (physiological saline injections). Parasitemia and survival were monitored as described for infection with wild type AnTat1.1. Animals were humanely sacrificed when showing severe clinical signs or when the parasitemia reached 10^8^ parasites·mL^-1^.

### Nanoparticles uptake

NbAn33-chNPs (1 mL at 1 mg·mL^-1^) were labelled using the Alexa Fluor 594 Protein labeling kit (Invitrogen), in 5 mM KCl, 80 mM NaCl, 1 mM MgSO_4_, 20 mM Na_2_HPO_4_, 2 mM NaH_2_PO_4_, pH 7.4) at room temperature for 1 h. Labelled NP were washed three times by centrifugation at 14,000 g for 30 min and resuspended in 5 mM KCl, 80 mM NaCl, 1 mM MgSO_4_, 20 mM Na_2_HPO_4_, 2 mM NaH_2_PO_4_, 20 mM glucose, pH 7.4, at a final concentration of 1 mg·mL^-1^. Live bloodstream forms (10^6^ cells·mL^-1^) were incubated in 5 mM KCl, 80 mM NaCl, 1 mM MgSO_4_, 20 mM Na_2_HPO_4_, 2 mM NaH_2_PO_4_, 20 mM glucose, pH 7.4 (TDB) with Alexa 594 labelled NbAn33-chNPs (50 μg·mL^-1^) for 10 min at 37°C. NPs excess was removed by centrifugation at 4°C. Parasites were resuspended in TDB with tomato lectin-FITC conjugate (Sigma) at 20 μg·mL^-1^, incubated for either 2 or 10 min at 37°C and then fixed in 4% paraformaldehyde in PBS for 1 h at 4°C. Finally, trypanosomes were washed with PBS three times, spread on poly-L-lysine-coated slides, and mounted in DAPI-containing Vectashield medium (Vector Laboratories). For fluorescence microscopy analysis image acquisition was performed with a LSH 710 Confocal Microscope (Zeiss) and image analysis with ZEN 2012 (Zeiss) software.

### Genotyping

Genomic DNA was extracted using DNAzol reagent (Invitrogen), according to the manufacturer’s protocol. The *TbAT1/P2* complete open reading frame (TriTrypDB Tb927.5.286b) was amplified by PCR using the specific primers AT1F (5’ ATG CTC GGG TTT GAC TCA GC 3’) and AT1R (5’ CTA CTT GGG AAG CCC CTC AT 3’) [52]. The PCR was performed with AccuTherm DNA polymerase (Genecraft Germany) with the followings parameters: 1 cycle of 95°C for 2 min and 35 cycles of (95°C, 50 s; 50°C, 50 s; 72°C, 2.5 min). PCR products were run on a 1% agarose gel and purified on a silica membrane column (Nucleospin gel and PCR clean up, Macherey Nagel). The purified PCR products were directly sequenced with the same primers as used for PCR amplification.

The *AQP2* (TriTrypDB Tb927.10.14170) and *AQP3* complete genomic sequences (TriTrypDB Tb927.10.14160) were amplified with AccuTherm DNA polymerase using the forward primer AQP2/3F (5' GCT CCA GAA AAT CAG AAT GC 3') and the reverse primers AQP2R (5' GCG AAG GGT ATT GAC GGT TA 3') and AQP3R (5' GTG CCA CAC TAA TCT GCA TG 3'), respectively. The PCR conditions were: 1 cycle of 95°C for 2 min and 35 cycles of (95°C, 50 s; 47°C, 50 s; 72°C, 2.5 min). The PCR products were purified and sequenced. Two internal forward primers were designed to confirm the chimeric *AQP2/AQP3* sequence; AQP2Fi (5' GAG CGG TGG GAT GCA GAT G 3') and AQP3Fi (5' CGC CAC GGT TAT CAT TGA TGG G 3'). TbAQP3/TbAQP2 sequence was submitted to GenBank; accession number KR059026. The complete *AQP2-AQP3* locus was amplified using the forward primer SEC13 (5’ CAAAATCAGCGGGTTCACTG 3’) located at the end of the SEC13 gene (TriTrypDB Tb927.10.14180) and the reverse primer AQP3R. The PCR was performed with AccuTherm DNA polymerase with the followings parameters: 1 cycle of 95°C for 2 min and 35 cycles of (95°C, 50 s; 50°C, 50 s; 72°C, 6.5 min). PCR products were run on a 0.8% agarose gel and purified on a silica membrane column (Nucleospin gel and PCR clean up, Macherey Nagel). The purified bands were directly sequenced with different combinations of the above primers to cover the complete sequence of the locus.

### Real time quantitative reverse transcription PCR (real-time qRT-PCR)

Trypanosomes were harvested in 1 mL TRIzol reagent (Invitrogen) and total RNA was isolated following manufacturer’s protocol. First strand cDNA synthesis was performed using SuperScript III Reverse Transcriptase (Invitrogen) and Oligo dT20 as primer. Quantitative PCR amplification was performed using SYBR Green Master Mix (Bio Rad). The levels of Tb*AT1* mRNA were normalized against actin mRNA and the relative quantification was calculated by the ΔΔC_T_ method. Primers for *TbAT1* were AT1_863F (5’ CGA CTT CGC AGC AGA TGT TAA TG 3’) and AT1_956R (5’ CGG CAG GGT AGA CGA GAA ATG 3’), For actin the primer used were ACT206F (5’ AAT GAG CAA GCG ATG ATG GG 3’) and ACT348R (5’ GCA ACT CGT TAT AGA AGG TAT GG 3’) [53]. Thermal cycling was carried out as follows: 1 cycle of 95°C for 5 min and 40 cycles of (95°C, 15 s; 60°C, 60 s).

## Supporting Information

S1 Fig Release of pentamidine from NbAn33-pentamidine-chNPs as a function of the incubation time and pH. (TIF)

S2 Fig IC_50_ curves analysis. Error bars, S.D. from 3–9 independent experiments. Testing pentamidine, pentamidine-chNPs (pentamidine-loaded PEGlycated chitosan nanoparticles), NbAn33-pentamidine-chNPs (nanobody-coated pentamidine-loaded PEGlycated chitosan nanoparticles), NbAn33-chNPs empty (nanobody-coated PEGlycated-chitosan nanoparticles) and chNPs-empty (PEGlycated chitosan nanoparticles).
(TIF)

S3 Fig Analysis of AQP2/3 locus from wild type AnTat1.1 and *Tb*R25 showing the structure of both alleles in the latter. Top, PCR amplification strategy of *AQP2*, *AQP3* and the entire locus. Bottom, nucleotide sequence of *AQP3/AQP2* chimeric gene (Genbank accession number: KR059026). Bold indicates 453 nucleotides corresponding to *AQP3* (Aquaglyceroporin 3) (TriTrypDB Tb927.10.14160); underlying indicates 462 nucleotides corresponding to *AQP2* (Aquaglyceroporin 2) (TriTrypDB Tb927.10.14170); grey indicates primers binding site; numbers indicate the position in chromosome 10 (Tb927_10_v5; strain 927 chromosome 10, version 5).
(TIF)

S1 Table Effect of formulation conditions on nanoparticles parameters. (DOCX)

S2 Table Blood compatibility assays. Blood compatibility of pentamidine unloaded PEGylated chitosan nanoparticles (chNPs-empty), pentamidine-loaded PEGlycated chitosan nanoparticles (pentamidine-chNPs), nanobody-coated PEGlycated-chitosan nanoparticles (NbAn33-chNPs) and nanobody-coated pentamidine-loaded PEGlycated chitosan nanoparticles (NbAn33-pentamidine-chNPs) in terms of hemolysis (%), platelet activation (sP-selectin release, ng·mL^-1^), complement activation (C3a release: C3a desArg, ng·mL^-1^), and plasma recalcification time (T_1/2_ max, min). For drug delivery applications hemolysis value ≤ 2.9% is considered hemocompatible.
(DOCX)
