# Supplementary figures and images for: Specific Cell Targeting Therapy Bypasses Drug Resistance Mechanisms in African Trypanosomiasis
Source: PLoS Pathog. 2015 Jun 25;11(6):e1004942. doi: 10.1371/journal.ppat.1004942 (PMC4482409; doi:10.1371/journal.ppat.1004942)

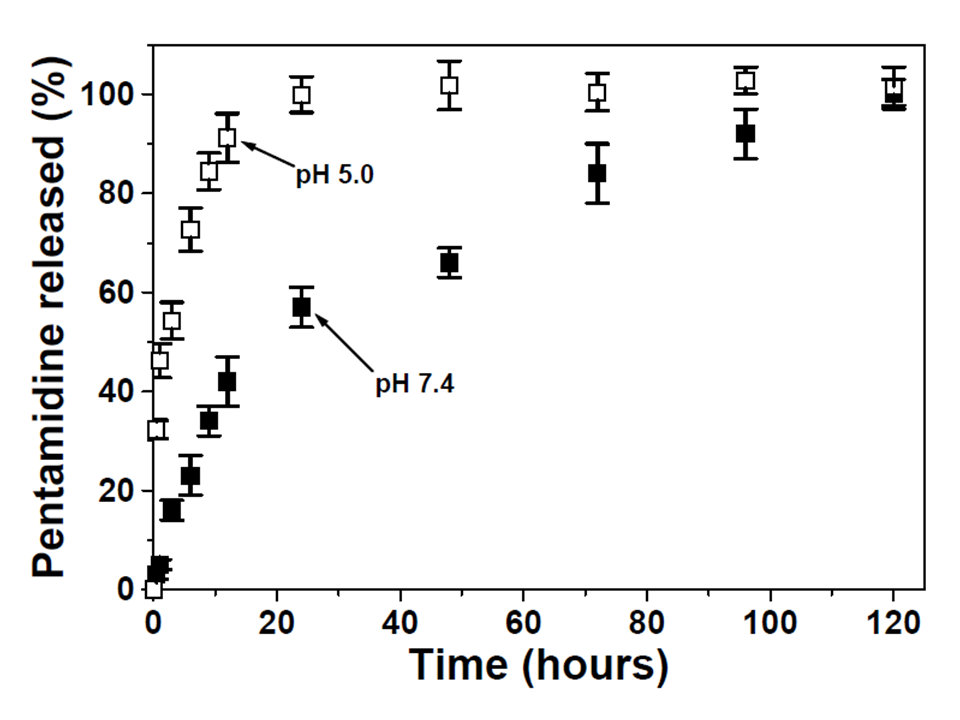

Supplement: S1 Fig — (TIF) [file ppat.1004942.s001.tif]

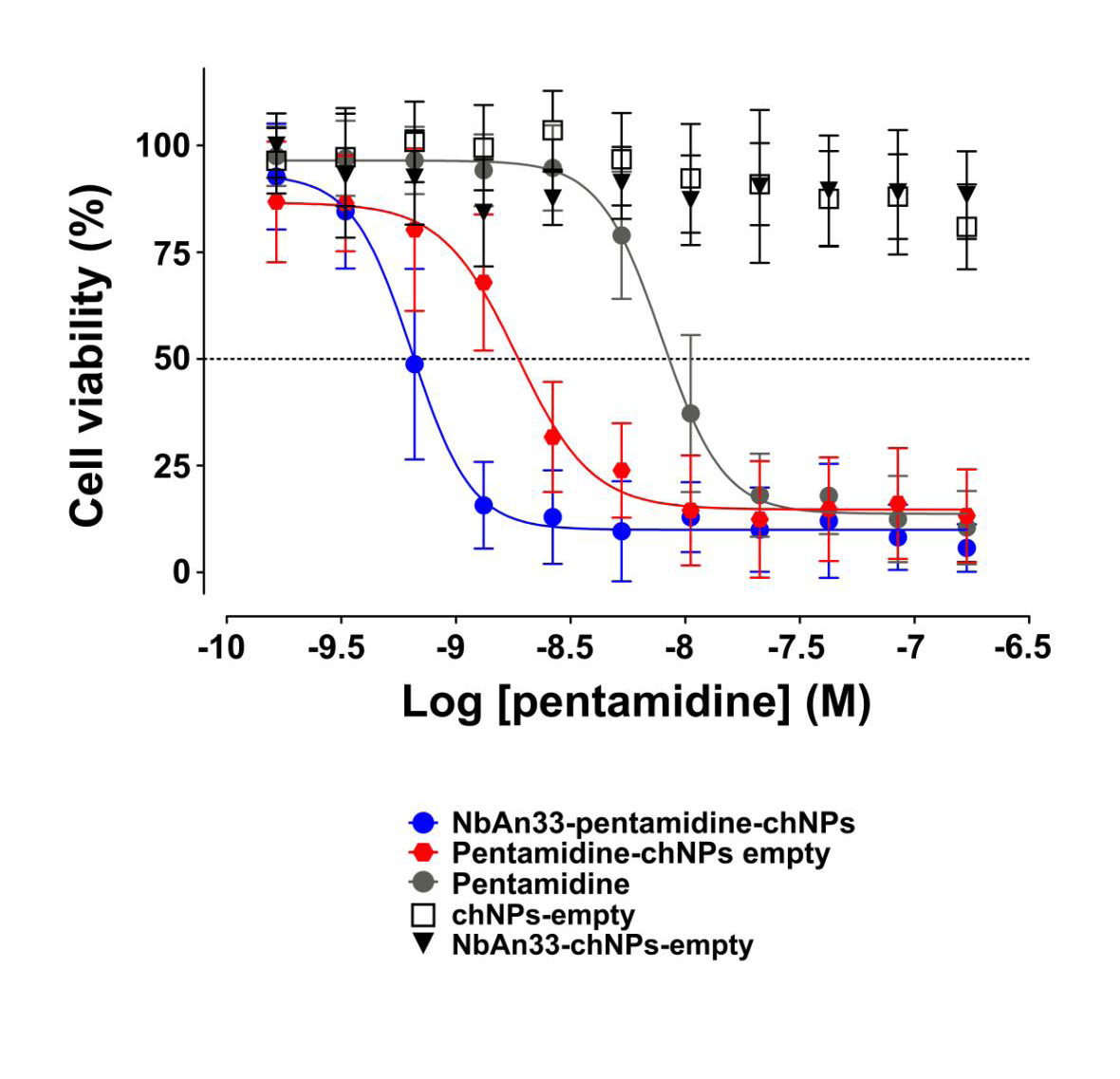

Supplement: S2 Fig — Error bars, S.D. from 3–9 independent experiments. Testing pentamidine, pentamidine-chNPs (pentamidine-loaded PEGlycated chitosan nanoparticles), NbAn33-pentamidine-chNPs (nanobody-coated pentamidine-loaded PEGlycated chitosan nanoparticles), NbAn33-chNPs empty (nanobody-coated PEGlycated-chitosan nanoparticles) and chNPs-empty (PEGlycated chitosan nanoparticles). (TIF) [file ppat.1004942.s002.tif]

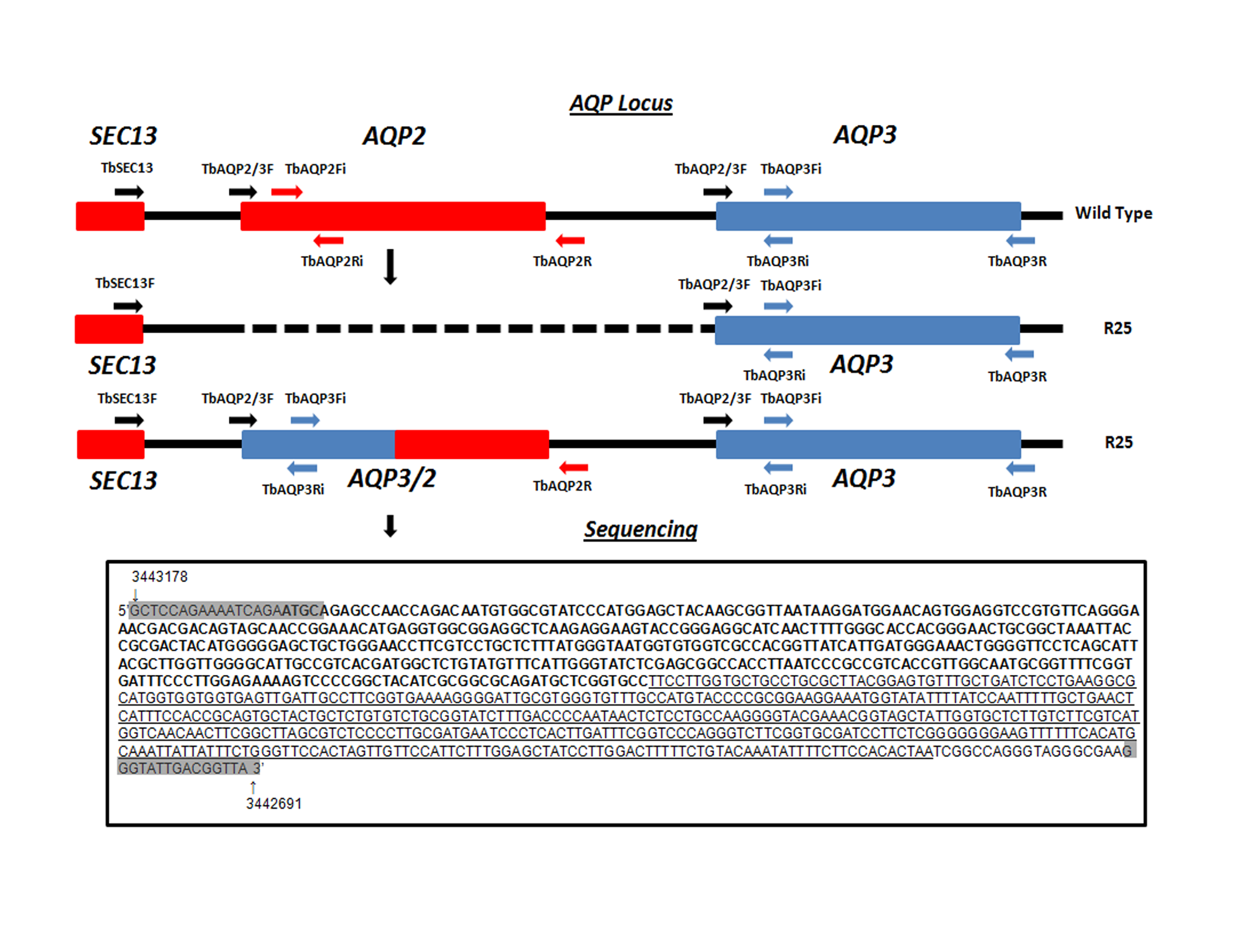

Supplement: S3 Fig — Top, PCR amplification strategy of AQP2, AQP3 and the entire locus. Bottom, nucleotide sequence of AQP3/AQP2 chimeric gene (Genbank accession number: KR059026). Bold indicates 453 nucleotides corresponding to AQP3 (Aquaglyceroporin 3) (TriTrypDB Tb927.10.14160); underlying indicates 462 nucleotides corresponding to AQP2 (Aquaglyceroporin 2) (TriTrypDB Tb927.10.14170); grey indicates primers binding site; numbers indicate the position in chromosome 10 (Tb927_10_v5; strain 927 chromosome 10, version 5). (TIF) [file ppat.1004942.s003.tif]
