# Supplementary material for: Specific Cell Targeting Therapy Bypasses Drug Resistance Mechanisms in African Trypanosomiasis
Source: PLoS Pathog. 2015 Jun 25;11(6):e1004942. doi: 10.1371/journal.ppat.1004942 (PMC4482409; doi:10.1371/journal.ppat.1004942)
Supplement: S1 Table — (DOCX) [file ppat.1004942.s004.docx]

**Table S1.** Effect of formulation conditions on nanoparticles parameters

| **[Pentamidine] (M)** | **Copolymer chitosan-*g*-PEG] (%, w/v)** | | **[Pluronic F-68] (%, w/v)** | **Size (nm)** | **Pentamidine entrapment efficiency (%)** | **Pentamidine loading (%)** | **Production performance (%)** | ***ζ* (mV)** |
| --- | --- | --- | --- | --- | --- | --- | --- | --- |
| 0 | | 1 | 1 | 132 ± 27 | – | – | 96 ± 4 | 15 ± 3 |
| 10^-5^ | | 1 | 1 | 134 ± 16 | 9 ± 3 | 0.003 ± 0.001 | 97 ± 3 | 16 ± 2 |
| 10^-4^ | | 1 | 1 | 126 ± 22 | 26 ± 3 | 0.088 ± 0.011 | 94 ± 5 | 18 ± 3 |
| 10^-3^ | | 1 | 1 | 137 ± 18 | 48 ± 6 | 1.634 ± 0.204 | 95 ± 2 | 14 ± 4 |
| 10^-2^ | | 1 | 1 | 134 ± 25 | 67 ± 4 | 22.808 ± 1.362 | 97 ± 2 | 19 ± 5 |
| 10^-2^ | | 1 | 0 | Macroaggregates | 3 ± 1 | 1.021 ± 0.341 | 4 ± 1 | 16 ± 3 |
| 10^-2^ | | 1 | 0.5 | 133 ± 24 | 64 ± 5 | 21.787 ± 1.702 | 95 ± 3 | 14 ± 3 |
| 10^-2^ | | 1 | 2 | 144 ± 22 | 66 ± 4 | 22.468 ± 1.362 | 96 ± 2 | 15 ± 4 |
| 10^-2^ | | 0.5 | 1 | 138 ± 25 | 62 ± 3 | 42.212 ± 2.042 | 94 ± 3 | 17 ± 3 |
| 10^-2^ | | 2 | 1 | 142 ± 19 | 65 ± 2 | 11.064 ± 0.341 | 96 ± 4 | 18 ± 2 |
