# Supplementary material for: Specific Cell Targeting Therapy Bypasses Drug Resistance Mechanisms in African Trypanosomiasis
Source: PLoS Pathog. 2015 Jun 25;11(6):e1004942. doi: 10.1371/journal.ppat.1004942 (PMC4482409; doi:10.1371/journal.ppat.1004942)
Supplement: S2 Table — Blood compatibility of pentamidine unloaded PEGylated chitosan nanoparticles (chNPs-empty), pentamidine-loaded PEGlycated chitosan nanoparticles (pentamidine-chNPs), nanobody-coated PEGlycated-chitosan nanoparticles (NbAn33-chNPs) and nanobody-coated pentamidine-loaded PEGlycated chitosan nanoparticles (NbAn33-pentamidine-chNPs) in terms of hemolysis (%), platelet activation (sP-selectin release, ng·mL-1), complement activation (C3a release: C3a desArg, ng·mL-1), and plasma recalcification time (T1/2 max, min). For drug delivery applications hemolysis value ≤ 2.9% is considered hemocompatible. (DOCX) [file ppat.1004942.s005.docx]

**Table S2.**Blood compatibility assays

| Sample* | Hemolysis (%) | sP-selectin release (ng/mL) | C3a desArg (ng/mL) | T1/2max (min) |
| --- | --- | --- | --- | --- |
| chNPs-empty | 1.7 ± 0.3 | 109 ± 6 | 287 ± 11 | 11.7 ± 1.3 |
| Pentamidine-chNPs | 2.4 ± 0.5 | 99 ± 11 | 291 ± 6 | 12.3 ± 0.7 |
| NbAn33-chNPs | 2.1 ± 0.2 | 96 ± 7 | 289 ± 7 | 11.9 ± 1.4 |
| NbAn33-pentamidine-chNPs | 2.2 ± 0.7 | 106 ± 4 | 293 ± 9 | 10.6 ± 1.3 |
| Control (PBS solution) | 0 | 103 ± 12 | 288 ± 13 | 10.5 ± 1.3 |

*Blood compatibility of pentamidine unloaded PEGylated chitosan nanoparticles (chNPs-empty), pentamidine-loaded PEGlycated chitosan nanoparticles (pentamidine-chNPs), nanobody-coated PEGlycated-chitosan nanoparticles (NbAn33-chNPs) and nanobody-coated pentamidine-loaded PEGlycated chitosan nanoparticles (NbAn33-pentamidine-chNPs) in terms of hemolysis (%), platelet activation (sP-selectin release, ng/mL), complement activation (C3a release: C3a desArg, ng/mL), and plasma recalcification time (T ½ max, min). For drug delivery applications hemolysis value ≤ 2.9% is considered haemocompatible.
